# A construction classification system database for understanding resource use in building construction

**DOI:** 10.1038/s41597-022-01141-8

**Published:** 2022-02-09

**Authors:** Gursans Guven, Aldrick Arceo, Allison Bennett, Melanie Tham, Bolaji Olanrewaju, Molly McGrail, Kaan Isin, Alexander W. Olson, Shoshanna Saxe

**Affiliations:** 1 University of Toronto, Department of Civil and Mineral Engineering, Toronto, Ontario M5S 1A4 Canada; 2 University of Toronto, Centre for Analytics and Artificial Intelligence Engineering, Toronto, Ontario M5S 1A4 Canada

**Keywords:** Environmental impact, Sustainability, Civil engineering

## Abstract

The building sector is a voracious consumer of primary materials. However, the study of building material use and associated impacts is challenged by the paucity of publicly available data in the field and the heterogeneity of data organization and classification between published studies. This paper makes two main contributions. First, we propose and demonstrate a building material data structure adapted from UniFormat and MasterFormat, two widely used construction classification systems in North America. Second, the dataset included provides fine grained material data for 70 buildings in North America. The dataset was developed by collecting design or construction drawings for the studied buildings and performing material takeoffs based on these drawings. The ontology is based on UniFormat and MasterFormat to facilitate interoperability with existing construction management practices, and to suggest a standardized structure for future material intensity studies. The data structure supports investigation into how form and building design are driving material use, opportunities to reduce construction material consumption and better understanding of how materials are used in buildings.

## Abstract (summary)

**Table:** 

Measurement(s)	material quantities
Technology Type(s)	digital curation • material takeoffs
Factor Type(s)	building • building type • location
Sample Characteristic - Environment	anthropogenic environment
Sample Characteristic - Location	Canada • United States of America

Machine-accessible metadata file describing the reported data: 10.6084/m9.figshare.17209331

## Background & Summary

This paper presents a novel dataset and data structure to facilitate study of resource use in building design and construction. The rapid urbanization of our world is a resource intensive process that has resulted in the irreversible, unsustainable transfer of material from nature into our anthropogenic stock^1^. The 20^th^ century saw a rapid increase in material stocks accumulated in buildings and infrastructure^2^, a trend projected to accelerate through the 21^st^ century. Overall material flows (i.e. resource use and waste) must be reduced by a factor of four to ten to achieve sustainable development^3^. To improve material efficiency, we need to better understand how and for what purposes material accumulates in our built environment. A key challenge to better understanding the resource cycle in the construction, maintenance and demolition of buildings is the paucity of data publicly available on building materials. While in theory detailed information is available for each constructed building in the form of design drawings, quantity takeoffs and construction records; recording material use is generally not a priority in an industry pressed by the competing challenges of cost and speed. Further, there has historically been little overlap between industrial ecology and engineering project management research leaving a disconnect between those with construction records and expertise, and those most interested in material stocks and flows. This research draws on construction management-based data organization and material takeoff methods to present a novel dataset and data structure for building material use research. This work presents a novel level of data disaggregation facilitating the study of where in buildings and for what purposes materials are used.

This study builds on the earlier building material intensity (MI) databases by others^4–9^. MI databases have been developed for determining material quantities and embodied carbon dioxide of different building types^4^, establishing country-wide databases for residential and urban buildings^5,8,9^, creating an open database by aggregating material intensity data from the literature^6^, estimating building in use material stocks and estimating the future demand for construction materials and availability of waste materials^7,10^. While the pace of new urban construction material flow analysis (MFA) has been accelerating, there remains a lack of standardization and a lack of classification of materials based on their specific use across the available datasets; nearly all available data counts materials aggregated over the whole building. Further, in-house datasets are increasingly being developed (e.g. EC3 tool^11^, Athena Impact Calculator^12^, Building for Environmental and Economic Sustainability (BEES) software^13^); but publicly available data, broadly available to the research community, remain rare.

This study extends the earlier MFA database research to include more buildings and more geographies in time, increase the level of detail on where in the building (e.g. above or below ground) and for what purpose (e.g. slab vs. column) construction materials are being used. Much of the existing building MFA research and databases have focused on understanding existing material stocks and the potential for building material reuse and recycling from that stock. This work focuses on the ability to investigate in more depth how building form and design decisions influence material consumption and as such focuses on new(er) buildings that reflect current construction practices. The database also adds a mass of low/similar uncertainty data as all the included building materials were quantified by the same team, using the same methods and from late-stage design or for construction drawings. To facilitate comparison and collation with existing databases, a translation script is included in the data repository^14,15^ that converts the data herein to the more aggregated structure proposed by Heeren and Fishman (2019)^6^.

This study builds on past research in industrial ecology and MFA which assesses the changes in flows and stocks of materials within a system^16^. Built environment MFA have been used to inform research on circular economy, urban mining, design for material efficiency and understanding historical material use in cities^17–19^. Detailed material identification has also been done for individual buildings for the purpose of life cycle assessments^20,21^. To facilitate meaningful changes in the way buildings are designed, built, demolished and reused; the quantities or percentage of materials that go into specific building elements (e.g., substructure) are needed^5,22,23^. However, building materials have not been disaggregated into different building elements generally in past studies. To address this gap, we adapted an ontology based on Uniformat^24^ and MasterFormat^25^, two widely used construction classification systems in North America, to facilitate a highly disaggregate assessment of material use.

## Methods

The developed ontology provides detailed material information including for what purpose (e.g. interior walls, floors, columns) in the building the material is used. This facilitates the ability to conduct more specific investigations in parts/elements of the buildings. This is useful for assessing how a particular element is contributing to material use in the building and how it can be designed for material replacement (e.g. steel vs. concrete, concrete vs. wood) or material efficiency (e.g. hollow vs. solid columns, above vs. below ground). Utilizing standard construction classification systems also facilitates interoperability with existing uses within construction project management such as costing, estimating, planning and environmental assessment of construction, and suggests a standardized structure for future MI studies.

The dataset provided in this study currently includes material estimates for 70 buildings in North America (i.e. 67 in Toronto, Ontario, one in Winnipeg, Manitoba, and one in Richmond, British Columbia, Canada; and one in New York, USA). 44 of these buildings are single-family dwellings (SFDs). Of these, 40 are proposed new builds for 2020 and 2021, and four are proposed renovations. 18 are mid to high-rise buildings constructed between 1988 and 2020 with one currently under construction. Eight are laneway suites (i.e. Accessory Dwelling Units (ADU) that are located on the same lot with a main dwelling as a detached, semi-detached or other low-rise dwelling^26^) that are built in between 2019 and 2021. The methods for creating this dataset included three main steps: (1) gathering design or construction drawings for the buildings in the dataset, (2) carrying out material takeoffs to quantify the construction material for each building, and (3) organizing the material data in a construction classifications systems-based database (specifically UniFormat and MasterFormat style). The assumptions made during the material takeoff process and limitations of the study are discussed below.

### Gathering design or construction drawings

Design and construction drawings were collected from both public and private sources. Detailed information for the 44 single family homes and one laneway suite were available from the City of Toronto Committee of Adjustment (CoA)^27^ and were downloaded between July 2019 and July 2020. The building applications available through the CoA website are construction or renovation projects that are applying for variance to existing zoning by-laws, and as part of the application process, details on the proposed construction are posted for public comment. Common reasons for CoA applications include single family houses that are requesting increased building height (e.g. proposed height of 8.51 metres for the permitted maximum height of 7.2 metres), larger driveway width (e.g. proposed driveway width of 10.30 metres for the maximum permitted driveway width of 6.0 metres), or bigger lot coverage (i.e. portion of the lot covered by the building) (e.g. proposed lot coverage of 40% for the permitted maximum lot coverage of 33%). While, by definition, a building requiring CoA is an exception to local planning rules; applications to the CoA are an extremely common step in home construction in Toronto, Canada. In between the years of 2015 and 2019, the CoA received over 3,500 minor variance (e.g. small changes to building setback or parking requirements) applications per year^27^. Construction drawings for the seven laneway suites and all mid to high-rise buildings were provided by architects, engineers and owners involved in the design and construction of the respective buildings. All construction drawings were either directly exported as Autodesk AutoCAD .dwg files or scanned copies of PDF files that were developed using AutoCAD.

### Performing takeoffs for the quantification of building materials

Quantities of construction materials for each building were calculated using material takeoff methods as described in Pratt (2010)^28^. For the materials used in each building element, the details provided in the schedules and notes in the construction drawings were examined, the dimensions (e.g. length, width, height) of each building element were measured and/or elements were counted from the drawings and the quantities of materials were calculated accordingly. For the details that were not available in the construction drawings (e.g. bricks, stones, mortar, I-joists) external sources of information, such as local and national building codes and product brochures, were used. In addition, industry experts, such as structural engineers and estimators, were consulted for advice on building design practice, particularly for estimating the rebar ratios of mid to high-rise buildings. Drawings of the newly proposed SFDs were imported to Autodesk AutoCAD 2020 to measure the lengths and areas of each construction material for each building element. Drawings of the renovated buildings, laneway suites and mid to high-rise buildings were exported to the On-Screen Takeoff (OST) tool^29^, and OST (v 3.96.00.23 and v 3.98.2.39) and AutoCAD (Q.47.0.0 AutoCAD 2020) were used in parallel. The research team also investigated the use of 3D models, such as Building Information Models (BIMs), as an alternative takeoff process, however, the elements that are usually omitted in BIMs (e.g. reinforcement bars), the level of detail of the models in current practice and the reality that models are often developed and used for earlier stages of design (and often do not reflect the final building) currently present a challenge in terms of facilitating the quality and detail of data presented in this study. ﻿Th﻿e﻿ details, measurements and calculated volume and mass of each construction material were recorded in Microsoft Excel spreadsheets. Details of the material takeoff process and the characteristics of the buildings included in the dataset are described in the Technical Validation section.

### Creating a construction classifications systems-based database

A database structure was developed using UniFormat and MasterFormat^24,25^ for recording the materials (e.g. concrete, steel, masonry) and determining for what purpose the material is used in the building (e.g. substructure, superstructure, floors, walls). The two classifications complement each other^24^: UniFormat subdivides a facility by functional building elements, and MasterFormat subdivides it by the materials used. Where insufficient specification exists within Uniformat and MasterFormat to clearly identify the building element and material; additions were made (more detail in online-only Table 1 below).

UniFormat (A Uniform Classification of Construction Systems and Assemblies) is mainly used for cost estimates and organized in a way that arranges construction information based on the functional characteristics of building elements which are physical parts of a facility (e.g. shell, superstructure, substructure)^24,30^. The original UniFormat classification was developed jointly by the General Services Administration (GSA) and the American Institute of Architects (AIA) in the early 1970s. The latest version of UniFormat was published in 2010 and is referenced in this study. MasterFormat is used as a specifications-writing standard for organizing construction information for commercial building design and construction projects in North America^25,30^. MasterFormat is focused on construction products and activities, and divides a building in terms of the related results achieved in a stage of construction (e.g. production, maintenance, demolition), which can be identified by the construction resources used (e.g. concrete, metals, wood), or the trade that was involved (e.g. earthwork, masonry)^24^. It has been used in bidding and specifications since 1960s^25,30^ and the 2020 version of MasterFormat is referenced in this study. Both UniFormat and MasterFormat are used to arrange and organize building information throughout the whole life cycle of buildings (i.e. planning, design, construction, operation, and maintenance), and this work makes use of the design and construction modules.

UniFormat has nine categories (i.e. *A-Substructure*, *B-Shell*, *C-Interiors*, *D-Services*, *E-Equipment and Furnishings*, *F-Special Construction and Demolition*, *G-Building Site Work*, and *Z-General*) further divided into four hierarchical levels (Levels 1 to 4) with increasing specificity of purpose^24^. An example breakdown of an element can be: Shell (Level 1), Superstructure (Level 2), Floor Construction (Level 3), Floor Structural Frame (Level 4). An additional Level 5 is added to UniFormat in this study to specify some structural elements in the takeoff that would otherwise be unclear (e.g. floor girders and beams) (more detail in online-only Table 1 below). With the focus on building material quantities, this dataset and data structure is so far limited to six Level 1 categories: 1) *Substructure*, 2) *Shell*, 3) *Interiors*, 4) *Services*, 5) *Sitework*, and 6) *Special Construction and Demolition*:

- *Substructure* includes the foundations, slabs-on-grade, and subgrade enclosures,
- *Shell* includes superstructure elements and exterior enclosures,
- *Interior* consists of elements such as interior construction and interior finishes,
- *Services* include the components such as conveying, heating, ventilation, and air conditioning (HVAC),
- *Sitework* includes site preparation and improvement works, such as roadways, parking lots.
- *Special Construction and Demolition* includes special facility components, such as pools, and special function construction, such as sound and vibration control.

MasterFormat includes 44 Divisions, each representing a particular trade, or the construction resources used in a building^25^. The database structure presented calls on eight of these divisions, namely *3-Concrete*, *4-Masonry*, *5-Metals*, *6-Wood, Plastics, and Composites*, *7-Thermal and Moisture Protection*, *8-Openings*, *9-Finishes, and 32-Exterior Improvements*. These are the divisions dealing with the description of building materials that were quantified. Other divisions dealing with facility services (e.g. plumbing, HVAC), site and infrastructure (e.g. utilities) and process equipment (e.g. pollution control equipment) were not used for describing the building materials studied in this work. Under each division, MasterFormat provides more categorization based on the nature of the related construction work. For example, there is a *Cast-in-Place Concrete* category under the *Concrete* division; it is further divided into *Concrete Finishing*, then *Polished Concrete Finishing*, followed by *Polished and Dyed Concrete Finishing* sub-category. In the database, the top-level divisions, such as *Concrete*, are named Level 1 and the further subcategories are named MasterFormat Level 2 to 5 (e.g. *Cast-in-Place Concrete* Level 2, *Concrete Finishing* Level 3, *Polished Concrete Finishing* Level 4, and *Polished and Dyed Concrete Finishing* Level 5), where applicable. Level 5 in UniFormat and Level 5 in MasterFormat do not exist for every building material. In our data structure, Uniformat Level 1 to 4, and Masterformat Level 1 are mandatory (i.e. they require a value to be entered). The other sublevels are optional and can be used to communicate more specificity on the building element or material. In the dataset, all materials have at least Level 1 through 4 in UniFormat, and most of the materials have Level 1 through Level 3 in MasterFormat.

As explained above, each building material in the database is categorized by UniFormat levels (e.g. *Substructure*, *Foundations*, *Standard Foundations*, *Wall Foundations*, *and Continuous Footings*) and MasterFormat levels (e.g. *Concrete*, *Cast-in-Place Concrete*, *Structural Concrete*, *and Lightweight Structural Concrete*) to describe its function and material, respectively. Accordingly, details of each building’s construction are finely recorded with increasing detail through the use of nested levels. Through aggregation to a higher level, the data structure allows aggregation for material (e.g. amount of concrete, or cast-in-place concrete, or structural concrete, or lightweight structural concrete), for function (e.g. substructure, or foundations, or continuous footings) and a combination of both (e.g. amount of lightweight structural concrete used in the substructure, or amount of concrete used in the foundations).

**Fig. 1:**
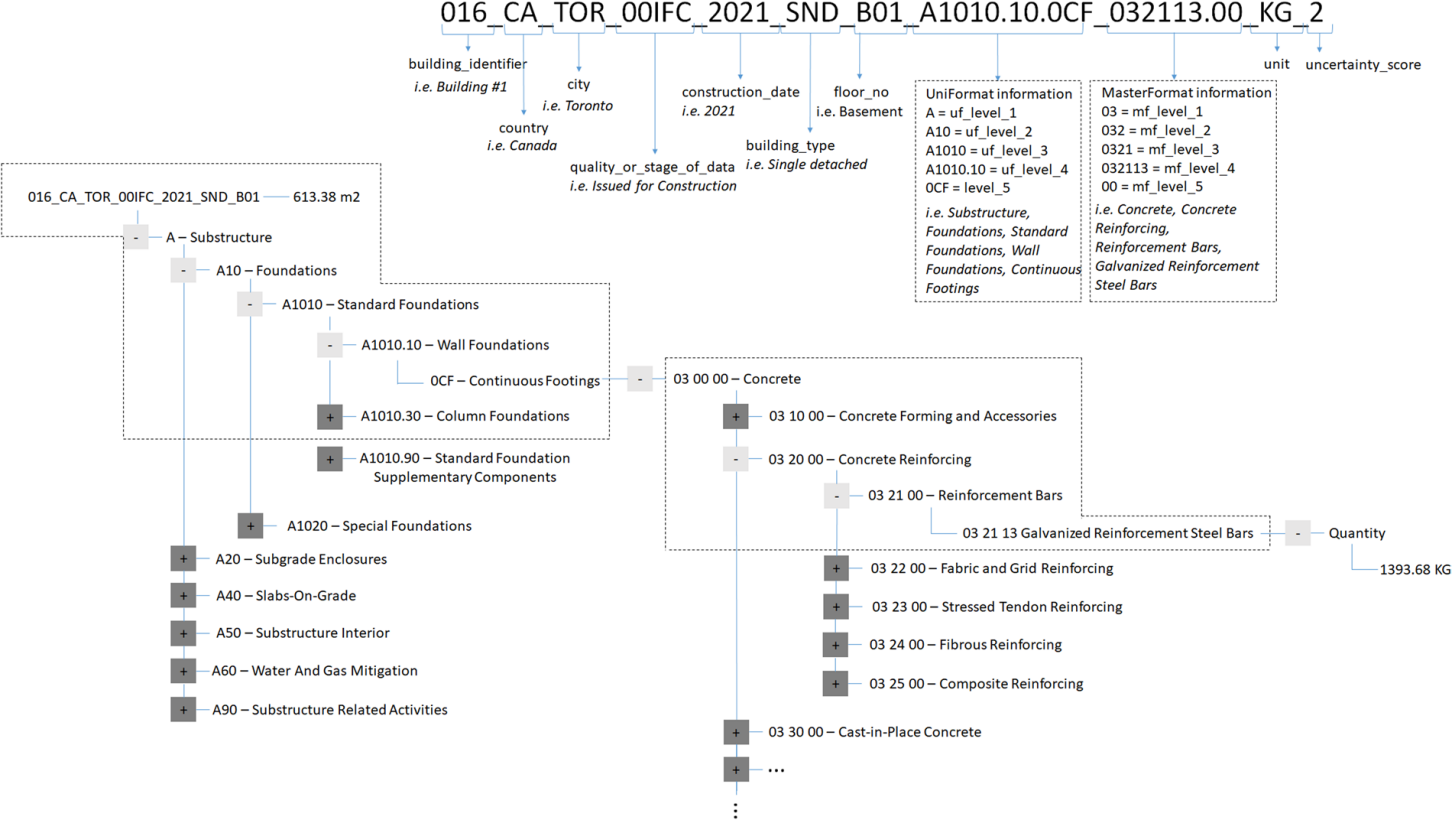
Sample data record 016_CA_TOR_00IFC_2021_SND_B01_A1010.10.0CF_032113.00_KG_2 and its tree view.

Figure 1 demonstrates how buildings and data entries are coded within the database and a sample approach for aggregating by material and function. This example is for a sample data record (i.e. 016_CA_TOR_00IFC_2021_SND_B01_A1010.10.0CF_032113.00_KG_2) that corresponds to the amount of concrete reinforcement in the continuous footings of one building. More specifically, this is a record of the amount of galvanized reinforcement steel bars used in the continuous footings of the case study building number 16 that is expected to be completed in 2021 in Toronto, Canada, and shows that this information is based on the issued for construction drawings. This example data record has an uncertainty score of 2, meaning that the material quantification is based on the original building drawings and details (e.g. specifications, notes, legends). In addition, for each building, the related gross floor area (GFA) of the building (i.e. 613.38 m^2^) is included in the data record, along with the amount of construction material that was calculated through material takeoff (i.e. 1,393.68 kg). Figure 1 illustrates the variables in each record and the tree diagram shows how this information is stored within the database structure. The structure is designed to facilitate simple data exploration, collation and analysis using computer code. Sample exploratory codes are included in the data repository^14,15^ which include calculations of material intensity in a building and queries on material uses for specific functions in buildings as well as the average amount of material used in a particular type of building (e.g. apartment buildings, single family dwellings) in a particular part of the building (e.g. interior, exterior, below ground). The sample code also shows how each building element category described in UniFormat contributes to the total material intensity. Sample calculations are also provided to calculate further descriptors of the buildings, for example a building’s climate zone based on its location and the number of floors relative to the ground (e.g. above ground, ground, below ground and roof).

Below is the detailed explanation of the variables for the data records in the database. A data record that consists of a code that has fifty-seven digits is created for takeoff within each building. The variables are detailed below, each of which are separated by an underscore (_) in the data record code:

- building_identifier is the first three digits in the data record code and is unique for each building in the dataset (e.g. 001, 002, etc.).
- country (two digits) is the country where the building is located. ISO 3166–1 alpha-2 code^31^, the two-letter country codes defined in ISO 3166–1, is used to identify the country name in the database structure.
- city (three digits) is the city where the building is located. The first three letters of the city name are used to represent the city in the database (e.g. TOR for Toronto). When new city names are added in the database, users should ensure they are using a unique identifier with their country. If the first three letters are already taken, users should choose other letters from the city name.
- quality_or_stage_of_data (five digits) communicates the quality or stage of building drawings, demonstrating the level of completion of construction documents, such as Issued for Construction (00IFC) or Issued for Building Permit (0IFBP).
- construction_date (four digits) is the year (or projected year) of completion. For renovated buildings, construction date is the year the building was originally constructed.
- building_type (three digits) shows the type of building that is quantified (e.g. single detached (SND), commercial (OFF), institutional (INS)). “R” in the building type code indicates that the material takeoffs were completed from drawings during renovation of the building (e.g. SNR for renovated single detached house).
- floor_level (three digits) describes the floor or part of building where the material is placed. The code designated for roof is 00R, while 999 represents the whole building, for example in the case of cladding materials covering the surface of the building. Underground floors are named based on purpose of use (i.e. basement or parking), and basement is denoted with letter B (e.g. B01) while parking is denoted with letter P (e.g. P02). Foundation is 00F, ground floor is 000, mezzanine floors are denoted with letter M (e.g. M00), and above-ground floors are 002, 003, etc.
- UniFormat information is represented with the next 12 digits in the data record code. The first digit is the uf_level_1 code, and it is followed by two-digit uf_level_2 code, followed by two-digit uf_level_3 code, followed by a dot, followed by two-digit uf_level_4 code, followed by a dot, and followed by three-digit uf_level_5 code, or 000 if there is no Level 5.
  - For example; A1010.30.0SF (A = *Substructure* = uf_level_1, A10 = *Foundations* = uf_level_2, A1010 = *Standard Foundations* = uf_level_3, A1010.30 = *Column Foundations* = uf_level_4, 0SF = *Spread Footings* = uf_level_5).
- MasterFormat information is the next nine digits in the data record code, first two digits being the mf_level_1 code, followed by one digit mf_level_2 code, followed by one-digit mf_level_3 code, followed by two-digit mf_level_4 code, followed by a dot, and followed by two-digit mf_level_5 code, or 00 if there is no Level 5.
  - For example, 033116.00 (03 = *Concrete* = mf_level_1, 033 *Cast-in-Place Concrete* = mf_level_2, 0331 = *Structural Concrete* = mf_level_3, 033116 *Lightweight Structural Concrete* = mf_level_4, 00 = mf_level_5).
- The next two digits of the data record is the unit. For each data record, the amount of construction material that was calculated via material takeoff is expressed in terms of mass (i.e. kg). Two quantities are reported for each material takeoff, a minimum and a maximum. For materials where direct measurements were possible (e.g. a concrete column) or only one value was available (e.g. composite decking dimensions from a brochure) the two values are the same and represent a discrete number. For quantities calculated through assumptions (e.g. the percentage of steel rebar which was based on expert elicitation) the minimum and maximum are different and reflect some of the uncertainty in the estimate.
- uncertainty_score (one digit) communicates the uncertainty of the data sources used in the quantification of the material on a scale of 1 to 6 (more detail in online-only Table 2 below).
- For every building, Gross Floor Area (GFA) information is stored in the database. The GFA is the total floor area of all floor levels including the underground space, and contains the area taken by external walls, internal walls, columns, and partitions. The unit of measurement for the GFA is square meters.

Although UniFormat and MasterFormat have long been used in practice in construction project management and costing, there were some adjustments and additions needed to facilitate a clear description of what functions the materials serve in each building. Online-only Table 1 summarizes the changes made. Additions were kept to a minimum to retain the advantages of interoperability with other uses of UniFormat and MasterFormat. Specific changes made are: UniFormat Level 5 is added to specify the structural element that is subject to material takeoff (e.g. column supporting floors), and a new Level 2 category and its related subcategories to UniFormat are added to classify the below-grade interior elements that are not part of the foundation and distinguish from above grade internal walls (Online-only Table 1).

### Assumptions made during the material takeoff process and limitations

During the material takeoff process, data were compiled from drawings made by different firms with different levels of detail. In some cases, this necessitated using additional indirect data sources such as local building codes, as further explained in online-only Table 2 in the Technical Validation section. Similarly, if direct measurements were not provided on the drawings, dimensions of building elements were scaled off the drawings which reduced accuracy.

Due to the large amount of material in mid to high-rise buildings, the scope of the material takeoff in these cases has been limited the major structural elements, and cladding elements when there was enough detail available in the drawings to facilitate cladding takeoffs. This meant a focus on concrete and steel, and wooden elements in the case where the framing of the building is timber. These represent the majority of materials in such buildings^4^. The scope includes all the concrete within the building footprint and excludes concrete finishing, smaller architectural concrete detailing, and for some buildings, temporary works used to support excavation. For steel, bulk structural steel was estimated, which includes steel decks, most rebar, steel members; while exclusions include structural connection details and anchor bolts, block wall rebar, composite deck rebar, stair rebar, metal grates, steel from shoring, MEP hardware, fittings, furnishings, and architectural features. For smaller buildings (e.g. SFDs and laneway suites), the quantification of materials includes structural members, envelopes, interior partitions, and excludes all materials used for mechanical and electrical services, architectural detailing for exterior walls, and furnishing.

Across the dataset, the drawings were prepared by different companies with different styles of presentation and detail. In particular, the material takeoff process of the renovated buildings was more challenging, due to changes in building codes between time of initial construction and studied renovation, and the potential for additional unrecorded past renovations. For the renovated buildings, the original year of construction is recorded in CoA documents, and the extent of the current renovation is known. In some cases, determining if a building element was new or original was ambiguous (for example: windows). There were also some building elements not shown on the available drawings (e.g. floor sheathing, exterior and interior wall structure, roof structure). In these cases information (e.g. dimensions, spacing) were estimated from either the Ontario Building Code (OBC) or the National Building Code (NBC) and the assigned uncertainty score adjusted accordingly.

## Data Records

The database is publicly available on Zenodo^15^. The database is built in Microsoft Excel as a summary data sheet with one column per building. The data repository on Zenodo contains a supporting document that explains the variables in the database, guidelines for contributing to the database and the additions applied to UniFormat version 2010^24^. The structure of the guidelines follows Heeren and Fishman (2019)^6^. Online-only Table 1 in the Methods section in this paper provides the details on the additions and modifications applied to UniFormat. A translation code is also provided in the repository that aggregates the database into the database format developed by Heeren and Fishman (2019)^6^ to facilitate combination of the new data in this paper into this pre-existing MFA database. A sample code written in Python for querying the dataset is provided as part of the data repository^14,15^. The sample code provides examples of, and easier coding for, data extraction and the type of information/queries that can be obtained from the dataset. The proposed data structure enables obtaining additional detailed information about the buildings in the dataset, such as climate and seismic conditions information based on location, or number of floors in a building above or below ground. Also, the structural details per floor and for the main envelope describe the construction type. The sample code includes sample graphs including code to reproduce figures in the Technical Validation section, such as MI in different parts of the studied buildings.

## Technical Validation

The dataset currently consists of 70 buildings (i.e. 44 SFDs, 18 mid to high-rise buildings and eight laneway suites). This ratio is similar to the ratio in the city of Toronto between the high-rise and low-rise buildings^32^. This section provides an overview of the data, the construction periods of the buildings, and summarizes the MI of the buildings in the dataset and contribution of building element categories to the total MI. It also explains the methodology used to communicate the uncertainty of the calculated material quantities based on the data quality.

### Description of buildings

The database is made up of low-rise buildings (i.e. single family homes and laneway suites) and mid to high-rise buildings with a range of functions (i.e. commercial, mixed-use residential condominiums, educational). 40 out of 44 of the SFDs included in the dataset are newly proposed buildings. Five were under construction in the last quarter of 2019. The remaining 35 are expected to start construction in 2020 and finish in 2021. All studied SFDs are wood-framed structures with finished basements constructed from concrete.

The remaining four SFDs were renovated in 2020; two were constructed in the early 1900s; the other two in 1969. These buildings are also wood-framed structures with finished basements constructed from concrete. The renovations include two second floor additions, one front basement walkout, and one attached carport. Undocumented renovations that took place between the initial construction and the current renovation are not considered given a lack of data.

The eight laneway suites are wooden frame buildings with reinforced concrete substructures built between 2019 and 2021. All laneway suites have two floors, and from 1 to 5 bedrooms. Two have indoor garages, two have basements and no garages, and four have no basements one of which has an attached garage. CS00060 is the maximum allowable size and has a fully excavated basement with walkout stairs. All eight are completely detached from the primary dwelling on the lot.

The 18 mid to high-rise buildings included in the dataset are completed buildings and span a 30-year period, ranging from 1988 to 2020 (as dated on the construction drawings and construction schedules) and one currently under construction. The number of floors ranges from 3 to 62. The buildings were built with a range of primary above-grade framing types (i.e. steel, concrete, wood, and steel/concrete or steel/wood hybrid). Shallow foundations, i.e., spread and raft footings were used for nine of the 18 buildings; while deep foundations, more specifically reinforced concrete caissons to rock or belled caisson foundations, were used for the other nine buildings. 16 of the 18 buildings include multi-layered underground reinforced concrete basements ranging from 1 to 5 floors, which are mostly for underground parking and bicycle storage.

### Data analysis

To demonstrate potential uses of the dataset, the following analyses are conducted using the sample code written in Python^15^: (1) Total material intensity (kg/m^2^ gross floor area) calculated based on MasterFormat Level 1 categories, and (2) percentage of a material (i.e. concrete) used in different building element categories (described in terms of UniFormat Level 2) in all the buildings across the dataset and its detailed breakdown for superstructure (UniFormat Level 4 and 5). These show the potential of the database to facilitate the exploration of trends in material use for individual buildings and across buildings. The sample code that is provided in the data repository queries the database in different additional ways, such as to investigate total material mass or intensity in a building, use of a material for specific functions, and average material intensity of materials used per floor across the buildings. Other examples include calculating the average amount of material used in a particular building type (e.g. apartment buildings, institutional, commercial), and to show the contribution of building element categories to total material intensities based on UniFormat levels (e.g. Substructure vs. Shell).

**Fig. 2:**
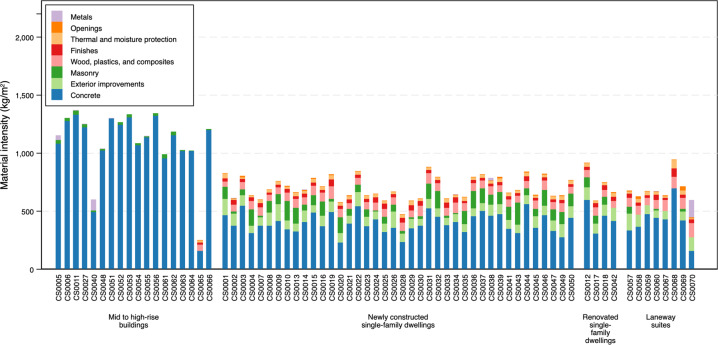
Total material intensity (kg/m^2^ gross floor area) broken down into MasterFormat Level 1 categories.

Figure 2 illustrates the total material intensity broken down into MasterFormat Level 1 categories. This illustrates the materials that contribute the most to the total material intensity across building types, as well as ranges in total material intensity per building and building type. This should be, however, interpreted in the context of the limitation of per m^2^ is a functional unit for buildings; per m^2^ does not account for the number of people who live in buildings or the full service provided^33^. The studied mid to high-rise buildings have the highest average total material intensity at 1,103 kg/m^2^ (*SD* = 281). The newly constructed single-family dwellings have an MI of 699 kg/m^2^ (*SD* = 96), renovated single-family dwellings at 730 kg/m^2^ (*SD* = 140) and laneway suites at 691 kg/m^2^, (*SD* = 108). The differences in the total material intensity across building types is due in large part to the high concrete and concrete reinforcement intensity of mid to high-rise buildings (*M* = 1,070 kg/m^2^, *SD* = 302) compared with low-rise buildings (newly constructed (*M* = 397 kg/m^2^, *SD* = 82), renovated single-family dwellings (*M* = 443 kg/m^2^, *SD* = 120) and laneway suites (*M* = 413 kg/m^2^, *SD* = 151)). This is consistent with past research that has found higher MI per m^2^ for high-rise buildings but lower MI per person as people live in less space in multi-unit buildings^34^. The materials quantified for the mid to high-rise were scope to major structural elements and cladding. As such, overall material intensity of these buildings is underestimated, the inclusion of other material categories (e.g. finishes, wood, plastics and composites, etc.) would increase the total materials and is an avenue for future research and expansion of the dataset.

**Fig. 3:**
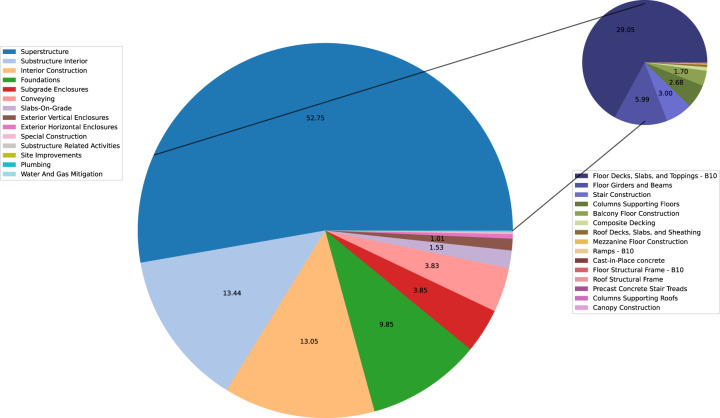
Percentage of total concrete across the entire dataset used in each building element category described as UniFormat levels (Level 2), and the breakdown of the Superstructure (UniFormat Level 2) category in terms of UniFormat Level 4 and Level 5 categories.

Further insights from the dataset include analysing the percentage of a particular material used in each building element category across the dataset. Figure 3 shows the percentage of concrete (as described in the Concrete MasterFormat Level 1 category, excluding Concrete Reinforcing and including Concrete Unit Masonry as described in MasterFormat Level 3) used in the building element categories according to UniFormat Level 2 (i.e. Superstructure, Foundations, Slabs-on-Grade). This illustrates that most of the concrete (52.8%) is used in the superstructure (such as the slabs, columns, beams, stairs, balconies, etc. that are above ground), followed by substructure interior (13.4%) that includes slabs, walls, columns and beams that are below ground; interior construction (13.1%) that includes above ground interior partitions and foundations category (9.9%) that include foundations such as column, wall and raft foundations. This highlights the potential to reduce material use significantly in buildings by reducing underground space. For example, changes in building design/massing that illuminated the need for interior walls underground (e.g. by eliminating parking levels with no reduction in foundation) could reduce material quantities by up to 13%.

A further breakdown of concrete used in the superstructure category, based on UniFormat Level 4 and 5, is also provided in Fig. 3. This illustrates that most of the above ground share of concrete is in slabs (29.1%), followed by floor girders and beams (6.0%), stairs (3.0%), columns supporting floors (2.7%), and balconies (1.7%).

### Uncertainty sources

The pedigree matrix originally developed by Weidema and Wesnaes^35^ is adapted to describe the uncertainty of the data sources used in the material quantification process. The pedigree matrix involves five data quality indicators: reliability, completeness, temporal correlation, geographical correlation, further technological correlation. In this work, the reliability metric data quality indicator originally developed by Weidema and Wesnaes^35^ is used to describe the acquisition sources and verification procedures used to gather the original building drawings. The reliability indicator pedigree matrix definitions are adapted to the material quantification process (online-only Table 2), and the uncertainties of each material takeoff in the dataset are described qualitatively. Online-only Table 2 summarizes the data sources used to quantify the building materials in the database and their uncertainties based on the adapted definitions.

Overall, the database adds a mass of low/similar uncertainty data to the existing MFA database research as all the included building materials were quantified by the same team, using the same methods and from late-stage design or for construction drawings. One caveat of this work is that all material estimates were based on drawings rather than onsite measurements. This adds a further level of uncertainty due to the commonplace use of more material onsite than predicted from drawings^36,37^. This is particularly the case for underground structures^38^. As such, the material numbers in the database likely underestimate (lower bound) the total materials involved in the case study buildings, as is common with all bottom-up approaches.

The different methods used for data collection for single family dwellings, laneway suites and mid to high-rise buildings also affect their uncertainties. While the single-family dwellings and one laneway suite were obtained from publicly available data and some details required referring to secondary sources (e.g. slabs-on-grade thickness from OBC), the complete sets of drawings of all mid to high-rise buildings and remaining laneway suites were directly obtained from the designers involved in their design and construction or builders or owners of the projects. In addition, the taller buildings drawing sets included more advanced specifications and details. To present the uncertainty level score of one building using a single value, a weighted uncertainty level is calculated by weighting the uncertainty level of each material by the respective mass and aggregating these weighted values. Equation (1) illustrates the calculation of the weighted uncertainty level of a building named “b”.

1 $$Weighted\;uncertainty\;leve{l}_{b}={\sum }_{i=1}^{n}Uncertainty\;leve{l}_{i}\times \frac{Quantit{y}_{i}}{Total\;quantit{y}_{i}}$$

In Eq. 1, “*i*” denotes each material of building “*b*” and “*n*” denotes the total number of materials in building “*b*”. “Quantity_i_” is the mass of material “*i*” of building “*b*”, “*Total quantity*_*i*_” is the mass of building “*b*”, and finally, “*Uncertainty level*_*i*_” is the uncertainty level of material “*i*” of building “*b*”.

**Fig. 4:**
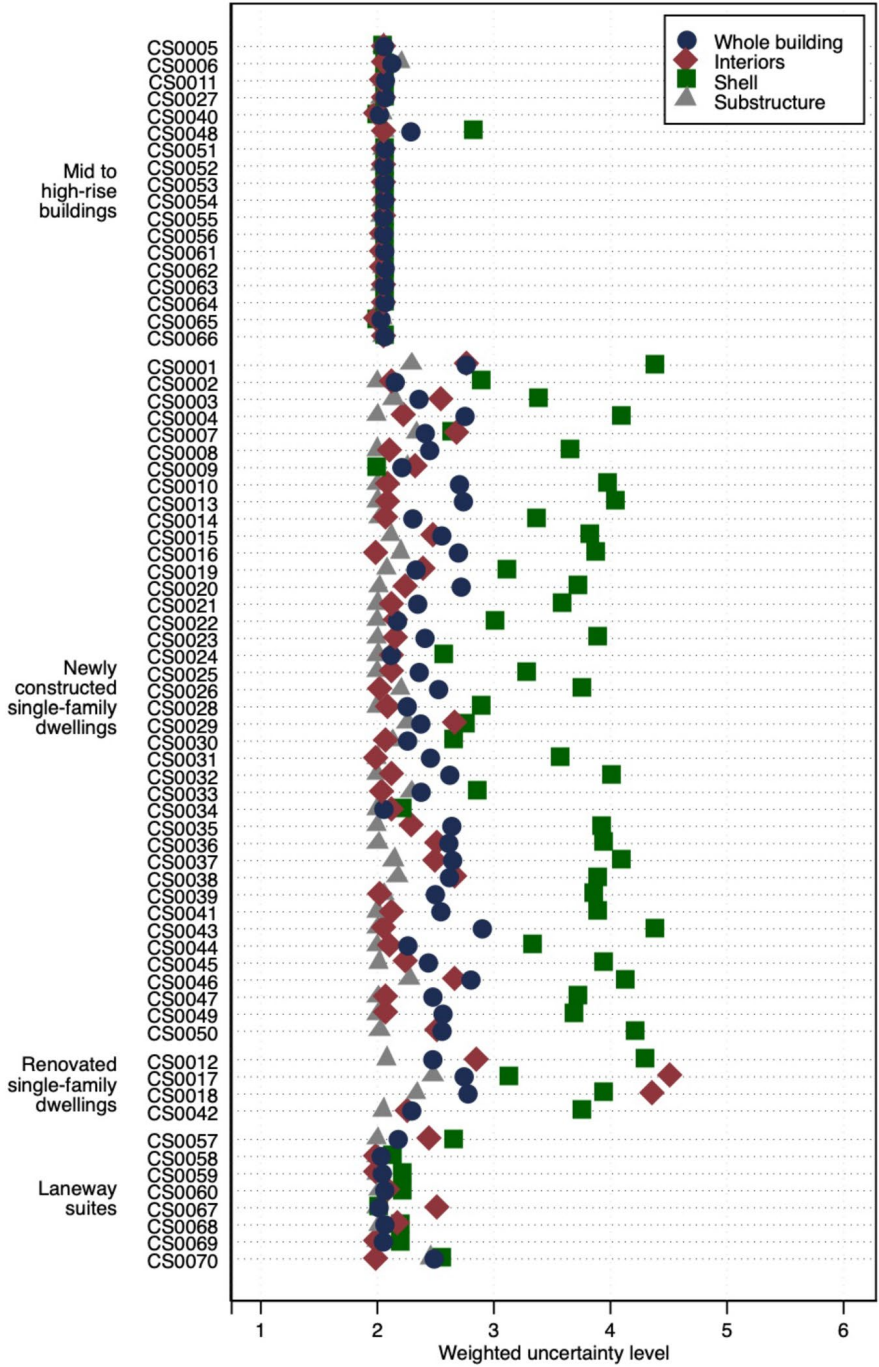
Weighted uncertainty level of the 70 buildings in the dataset, and the substructure, shell and interiors of each building.

The weighted uncertainty level for the three major building element categories of each building (i.e. substructure, shell, and interiors) are calculated using Eq. (1). Figure 4 illustrates the weighted uncertainty level of the buildings in the dataset grouped into mid to high-rise buildings, newly constructed single-family dwellings, renovated single-family dwellings and laneway suites. The weighted uncertainty level of the substructure, shell, and interiors of each building are also shown.

Focusing first on the single-family dwellings (newly constructed and renovated), the weighted uncertainty levels of the substructure and interiors are within the 2 to 3 range as majority of the data were from the construction drawings. The weighted uncertainty levels of the shell for most single-family dwellings (35 out of 44) is above 3, which is due to most data being sourced from construction material brochures. Although the weighted uncertainty level in the shell category is above 3, the overall weighted uncertainty levels were compensated by the low weighted uncertainty value of the substructure thanks to its large mass contribution (Fig. 4). The overall weighted uncertainty levels of the single-family dwellings, as well as the laneway suites, are between 2 and 3; the material intensity data is of high quality given that material takeoffs were based on building drawings and related codes. In the laneway suites, CS0057 building has slightly higher uncertainties in the interiors (2.5) and shell (2.7) categories compared to the rest of the laneway suites. These are due to the missing information in those building element categories, for instance related to resilient channels on floors and floor joists with inconsistent linear densities.

For the mid to high-rise buildings, 17 out of 18 (i.e. CS0005, CS0006, CS0011, CS0027, CS0040, CS0051, CS0052, CS0053, CS0054, CS0055, CS0056, CS0061, CS0062, CS0063, CS0064, CS0065,CS0066) buildings have whole building weighted uncertainty levels within 2 to 2.1 thanks to most of the data being sourced directly from the construction drawings. In contrast, the whole building uncertainty of CS0048 is slightly higher (2.3) due to the high weighted uncertainty level of the shell (2.8) as a result of much of the shell data being sourced from brochures, such as the composite decking and balconies.

## Usage Notes

The UniFormat and MasterFormat style database structure builds on existing construction management practices. It presents promise for interoperability with existing costing and construction management processes and allows for detailed communication of what materials are being used for where and for what purposes in a building. As with other proposed data structures, the adoption of a consistent ontology supports consistency of data over time and across buildings, and provides opportunities for contribution and further analysis by users.

As a result of the way the data is classified into building element categories and materials; the dataset can be used to assess how elements contribute to material use in buildings and how to better design for material replacement (e.g. steel vs. concrete, concrete vs. wood) in buildings and particularly to identify opportunities for material efficiency. From a building design perspective, understanding MI tied to different elements can inform changing the building design to reduce material needs while maintaining function (e.g. adjusting the balance of above ground vs. below ground floor space or adjusting the aspect ratio of the building). From a public policy perspective, the data can be used to evaluate the relative material consumption of different building forms and can be used to influence planning permissions (e.g. what buildings are allowed to be built where) and design codes.

The dataset presents a significant contribution to the body of knowledge on material use in buildings and a new set of publicly available data that contributes to the existing databases with buildings from North America (mostly Toronto, Canada). The construction approaches used in Toronto, Canada are indicative of those used across North America, and in new construction in many locations around the world, particularly where earthquake design requirements are low.

The dataset is publicly available on Zenodo^15^, and GitHub^14^ is used for the version control of the dataset. It is open to contribution, and therefore, guidelines to contribute are included in this paper and the supporting documents are provided on Zenodo and GitHub. The database structure based on UniFormat and MasterFormat style can be adjusted and updated by the users in accordance with the guidelines provided to facilitate data collection for new cities, new building elements (e.g. Electrical and Plumbing) and new levels of detail (e.g. adding new Level 5 elements to UniFormat) as needed. When new data is added to the dataset on GitHub, users will create copies of the repository (i.e. forks) and the changes will not affect the original dataset. To demonstrate the uses of the dataset and queries that can be performed on the dataset, a sample code written in Python is provided on GitHub^14^ and Zenodo^15^. A script that translates the database herein to the format of Heeren and Fishman (2019)^6^ is presented to facilitate fast combination of the detailed material/building function data in this dataset with the global MFA dataset developed by Heeren and Fishman (2019)^6^.

## Data Availability

No custom code was used in the production of this dataset. Use of existing software (Microsoft Excel) is described in the Methods and Data Records sections. The translation code that converts the dataset into the format developed by Heeren and Fishman (2019)^6^ and the sample code for querying the dataset are coded in Python. The dataset, translation script and sample code are made available in an open source repository^15^ and the Python packages used in the sample code are listed in the “requirements.txt” file (10.5281/zenodo.5576147).

## Online-only Tables

**Online-only Table 1:** List of additions and modifications made to UniFormat^24^ (version 2010).

Changes made	Why changes were made	Details
Level 5 was added	To name the specific structural element	Complete list of Level 5 elements and the corresponding value labels in brackets: • Continuous Footings [0CF] • Foundation Walls [0FW] • Spread Footings [0SF] • Column Piers [0CP] • Columns Supporting Floors [CSF] • Floor Girders and Beams [FGB] • Floor Trusses [0FT] • Floor Joists [0FJ] • Columns Supporting Roofs [CSR] • Roof Girders and Beams [RGB] • Roof Trusses [0RT] • Roof Joists [0RJ] • Parking Bumpers [0PB] • Precast Concrete Stair Treads [PCS] • Roof Curbs [0RC] • Exterior Wall Construction [EWC] • Composite Decking [CPD] • Cast-in-Place concrete [CIC] • Floor Structural Frame [FSF] • Associated Metal Fabrications [AMF] •Floor Construction Supplementary Components [FCS] • Roof Construction Supplementary Components [RCS] • Residential Elevators [0RE] • Vegetated Low-Slope Roofing [VLR] •Swimming Pools [SWP] • Excavation Soil Anchors [ESA] • Roof Window and Skylight Performance [RWS] • Rainwater Storage Tanks [RST] • Gray Water Tanks [GWT]
A50-Substructure Interior category and its subcategories were added under A-Substructure category	Within Uniformat 2010, there is no way to clearly label internal elements that are part of the substructure but not the foundation.	A50 - Substructure Interior (Level 2) • A5010 - Floor Construction (Level 3) ○ A5010.10 - Floor Structural Frame (Level 4) ○ A5010.20 - Floor Decks, Slabs, and Toppings (Level 4) ○ A5010.30 - Ramps (Level 4) • A5020 - Interior Partitions (Level 3) ○ A5020.10 - Interior Fixed Partitions (Level 4) ○ A5020.20 - Interior Partition Supplementary Components (Level 4) • A5030 - Ceiling Finishes ○ A5030.10 - Plaster and Gypsum Board Finish (Level 4)
A5010.10-Floor Structural Frame subcategory was added	To facilitate describing below grade structural frames	Structural elements required for support of floor construction within intermediate basement levels (i.e. columns, girders, beams, trusses, joists), while excluding slab-on-grade, exterior perimeter foundation walls, or foundation footings/deep caissons, etc.
A5010.20-Floor Decks, Slabs, and Toppings subcategory was added	To facilitate describing below grade floor construction	Structural slab, deck, and sheathing floor construction for intermediate basement level; housekeeping pads for mechanical, electrical, and similar equipment.
A5010.30-Ramps subcategory was added	To specifically describe which structural elements belong to this newly added category	Structural frame and decks, slabs, and sheathing for basement and below-grade ramp construction, while excluding slab-on-grade.
A5020.10-Interior Fixed Partitions subcategory was added	To specifically describe which elements belong to this newly added category	Interior enclosures and partitions which are fixed and secured in place within basement levels. Includes walls of concrete, and masonry, and wood and metal stud partitions with associated wall surfaces. Partitions may be load bearing or non-load bearing.
A5020.20-Interior Partition Supplementary Components subcategory was added	To specifically describe which substructure interior elements belong to this newly added below grade category	Sound isolation components, firestopping, and expansion control included with substructure interior partition elements.
A5030.10-Plaster and Gypsum Board Finish subcategory was added	To specifically describe which substructure interior elements belong to this newly added below grade category	Lathing including accessories and trim. Includes substructure interior plaster including veneer plaster, acoustical plaster, and plaster fireproofing. Includes sound barrier boards and blankets. Includes unfinished and prefinished gypsum board and gypsum backing board for other finishes. Includes gypsum board fireproofing.
Definition of the B1010.10-Floor Structural Frame category was modified	To delineate between above and below ground structural frames given the addition of A5010.10-Floor Structural Frame category	Definition of this category was modified to exclude construction within the basement. Updated definition reads: “Structural elements required for support of floor construction above grade.”
Definition of the B1010.20-Floor Decks, Slabs and Toppings category was modified	To delineate between above and below ground floor decks, slabs and toppings given the addition of A5010.20-Floor Decks, Slabs, and Toppings category	Definition of this category was modified to exclude construction within the basement. Updated definition reads: “Structural slab, deck, and sheathing floor construction above grade.”

**Online-only Table 2:** Uncertainty according to the reliability of the data sources used for quantification of building materials (definitions adapted from Weidema and Wesnaes, 1996).

Pedigree matrix indicator scores	Reliability indicator pedigree matrix definition	Definition adapted to material quantification process	Data sources used to quantify materials in the database		
			In newly proposed SFDs and laneway suites	In renovated buildings	In mid to high-rise buildings
1	Verified data based on measurements	Material quantification based on measurements performed on site, reflecting as-built conditions	N/A	N/A	N/A
2	Verified data based on measurements	Material quantification based on building drawings and details (e.g. specifications, notes, legends)	*From building drawings:* Majority of the building materials (e.g. used for the slabs-on-grade, foundations, sub-grade enclosures, floor construction, exterior walls, and interior partitions)	*From details:* Additional materials used for renovation, such as lumber, plywood, gypsum board, vapor barrier and insulation for framing the additional building floors, and concrete and steel for the slabs and foundation walls of the additional walkout and carport	*From building drawings:* Concrete material quantities By scaling from building drawings: Details such as heights and thicknesses of footing columns, underground columns, slabs, exterior walls, and interior walls, that were not mentioned explicitly in the notes and specifications
3	Verified data partly based on assumptions or non-verified data based on measurements	Material quantification based on trusted references for information not included in the building drawings (e.g. local and/or national building codes, brochures, literature)	*From OBC:* Thickness of the structural slabs-on-grade, size and spacing of rebars for structural slabs-on-grade, area density of mesh to structurally support stucco cladding, and cross-section of lumber used for roof construction in newly proposed SFDs	*From NBC*: Most of the materials that constitute the original buildings before renovation (e.g. concrete and vapor barrier for the concrete slab; vapor barrier, wood framing, and drywall for the basement; and brick veneer cladding)..	*From brochures and manufacturer catalogues:* Thicknesses of slabs for composite steel deck slabs^39^ and deep caissons or founding elevations of deep footings^40^. Steel structural framing for floor and decks^41^, mass of steel for the decks^42^, linear density of steel joists^43^, and steel cross-section^44,45^. *From quantity takeoff software:* Concrete for stairs in high-rise buildings
4	Non-verified data partly based on qualified estimates	Material quantification based on consultation with industry experts and estimators for information not included in the building drawings	N/A	N/A	Calculation of steel rebars in each structural concrete member were based on the rule of thumb ratios obtained from consultation with Canadian estimators and structural engineers
5	Qualified estimates (e.g. by industrial party)	Material quantification based on proxy data for information not included in the building drawings	*From other similar buildings:* Lumber for architectural posts for six buildings, rubber roof membrane for two buildings, and joists for two SFDs Wire mesh reinforcements in basement slabs for three buildings, resilient channel soundproofing in garage walls and ceilings for two buildings, TJI joist dimensions and linear densities for one building, polyurethane foam thickness for one building, wood furring on exterior walls for one building, roof membrane for one building, interior door thickness for one building, dimple membrane and rigid insulation for foundation wall of one building in the laneway suites category.	*From OBC:* Lumber and gypsum board used for exterior walls and interior partitions, and lumber used for roof structural frame during renovation.	N/A
6	Non-qualified estimate	N/A	N/A	N/A	N/A
